# Robust *in-silico* identification of cancer cell lines based on next generation sequencing

**DOI:** 10.18632/oncotarget.16110

**Published:** 2017-03-10

**Authors:** Raik Otto, Christine Sers, Ulf Leser

**Affiliations:** ^1^ Knowledge Management in Bioinformatics, Institute for Computer Science, Humboldt-Universität zu Berlin, Berlin, Germany; ^2^ Charité Universitätsmedizin Berlin, Institute of Pathology, Berlin, Germany; ^3^ DKTK, German Consortium for Translational Cancer Research, Partner Site, Berlin, Germany

**Keywords:** cancer cell lines, next-generation sequencing, cell line-identification, DNA-sequencing, data-heterogeneity and incompleteness

## Abstract

Cancer cell lines (CCL) are important tools for cancer researchers world-wide. However, handling of cancer cell lines is error-prone, and critical errors such as misidentification and cross-contamination occur more often than acceptable. Based on the fact that CCL today very often are sequenced (partly or entirely) anyway as part of the studies performed, we developed Uniquorn, a computational method that reliably identifies CCL samples based on variant profiles derived from whole exome or whole genome sequencing. Notably, Uniquorn does neither require a particular sequencing technology nor downstream analysis pipeline but works robustly across different NGS platforms and analysis steps. We evaluated Uniquorn by comparing more than 1900 CCL profiles from three large CCL libraries, embracing 1585 duplicates, against each other. In this setting, our method achieves a sensitivity of 97% and specificity of 99%. Errors are strongly associated to low quality mutation profiles. The R-package Uniquorn is freely available as Bioconductor-package.

## INTRODUCTION

Cancer Cell Lines (CCLs) are an essential tool for cancer research world-wide [1]. CCLs help to uncover cancer etiology and to study the mode-of-action of anticancer drugs. They are indispensable for functional investigation of proteins and pathways with much reduced ethical and legal issues compared to patient-derived tumor samples [1, 2]. However, CCLs are susceptible to misidentification and cross-contamination [1–8]; estimates regarding the extend of such problems in published scientific results range from 18% to 36% [9, 10]. A prominent example is MDA-MB-435, which was originally derived from the M14 melanoma CCL, yet later misclassified as a mammary-tissue CCL [11]. This error had wide-ranging, negative consequences because a number of research results were attributed to the wrong tissue-type. Since no universally accepted nomenclature system for CCLs exists [1, 8], researchers keep on inventing names of little discriminative power. For example, the CCL TT is a distinctively different CCL than T.T, but the similarity of both names makes mixing them up very easy. Meanwhile, high-impact journals require explicit verification of CCL integrity with respect to identity and absence of cross-contamination prior to publishing related research-results [1]. Overall, CCL sample-identification has become an integral part of CCL-based research.

The usual way of establishing the identity of a CCL sample under study (from now on called query sample q) is to compare it to CCLs whose identity is known (from now on called R, a library of reference samples) by experimentally comparing certain cell line specific features [1, 3, 5, 6, 8]. Established identification methods differ in the characteristic genomic entity that is compared between q and the samples in R. While Short Tandem-Repeat analysis (STR) compares counts of tandem-repeats [6], the Single-Nucleotide-Polymorphism Panel Identification Assay (SPIA) compares the zygosities of distinct diploid single-nucleotide polymorphisms (SNPs) [4]. Both methods require additional and costly experiments which do not contribute to the scientific goal of the original study. Furthermore, in all available methods the genotyping-technology – including the subsequently used software – applied to analyze the query q and to analyze the references R must be identical for achieving the expected accuracy. This implies access to the physical samples, which is difficult in large projects with numerous partners where often only information on samples or data generated from these is exchanged, but not the samples themselves.

At the same time, modern CCL-based research is increasingly based on high-throughput next generation sequencing (NGS [3, 12–14]). All major CCL sequencing project such as CCLE [2], CellMiner project [15], or COSMIC CLP [16], made extensive NGS-based data for characterizing their CCLs publicly available. It is a natural idea to use these profiles for identifying the origin of a given query sample within such a reference library (or within multiple libraries). However, typical NGS procedures do not extract the kind of genetic information necessary for STR or SPIA-based identification, as both methods require homogeneous and locus-specific genotype data, but these loci are often omitted from sequencing or filtered afterwards because they are assumed to be unrelated to the cancer itself. Furthermore, major chromosomal deletions, e.g. the common phenomenon of losing the Y-chromosome [17], can render usage of pre-defined genotypes impossible.

Thus, the information required for identification is not readily available. Even if it was, the effectiveness of STR and SPIA on lab- and project-specific NGS data sets were unclear. Both methods were evaluated only with homogeneous NGS profiles, i.e., references and query samples were sequenced using the same technologies, algorithms, and filtering methods; on top, these procedures require that the ploidy of the reference samples R matches the ploidy of the query sample q. Such a scenario of homogeneous, easily comparable NGS data sets is quite different from that typically found today, where different labs use different technologies, leading to heterogeneous NGS profiles. For instance, Hudson et al. compared the small missense variant calls accompanying identical CCLs (as defined by the creators of the reference libraries) between CCLE and COSMIC CLP and found them coinciding at only 43% [18]. A prominent case depicturing the extend of data-heterogeneity is the *ISHIKAWA-HERAKLION-02ER* CCL which has been DNA-genotyped by the Broad institute, finding 213 missense mutations, and the Sanger institute, which reported 52 pair-wise different missense mutations [18]. Causes for the data heterogeneity between large-scale sequencing projects are complex and include technical and design aspects. For example, sequencing of sub-clonal and aneuploid cancer-cell cultures may cause heterogeneous sequencing results [19]. Furthermore, studies differ in their aims and priorities, leading to different choices of algorithmic parameters and workflow designs which in turn can cause differing genotyping results even for the same CCLs [20].

Here, we present Uniquorn, a novel *in silico* approach for the robust and fast identification of CCLs within reference libraries based on their variant profiles. Uniquorn uses only NGS data and is based on the assumption that already today, most experiments on CCLs involve extensive sequencing. The algorithm is designed to compare variant profiles derived from a wide range of sequencing technology, quality, depth, and scope to make it useful for large and distributed research projects. Uniquorn was developed to addresses cases where neither STR nor SPIA can be applied, as both obligatorily require reliable SNP-calls and STR-profiles at specific loci for identification. Technically, Uniquorn is based on the computation of confidence-scores for the pairwise identity of the query sample to any sample from a reference library R, taking into account the prevalence of each variant in the library and a statistical assessment of the observed number of common variants.

We evaluated our algorithm on three high-profile CCL data sets with altogether 1988 reference samples, namely COSMIC CLP (1024), CCLE (904) and NCI-60 CellMiner (60). NGS profiles between these libraries are highly heterogeneous, because different laboratories created the data using different technologies and software and even covering partly different genomic regions [18]. SNP-based identification using the available data is impractical, as in two out of these three sets all SNPs were filtered to facilitate identification of driver mutations. Furthermore, neither of these data sets contains information on STRs. In such a rather difficult setting, Uniquorn achieves a sensitivity of 97% at a specificity of 99%. We also show that several pairs of cell lines which our method identifies as identical although they have different names indeed should be considered identical considering their extremely similar mutational profiles, and identify several candidates for cross-contamination of cell lines. Finally, we confirm a very low probability of random false positive hits by comparing all reference libraries’ CCLs with 1024 genomes of the 1000 genomes projects [21].

## RESULTS

### Weighting of small genomic variants

**Figure 1:**
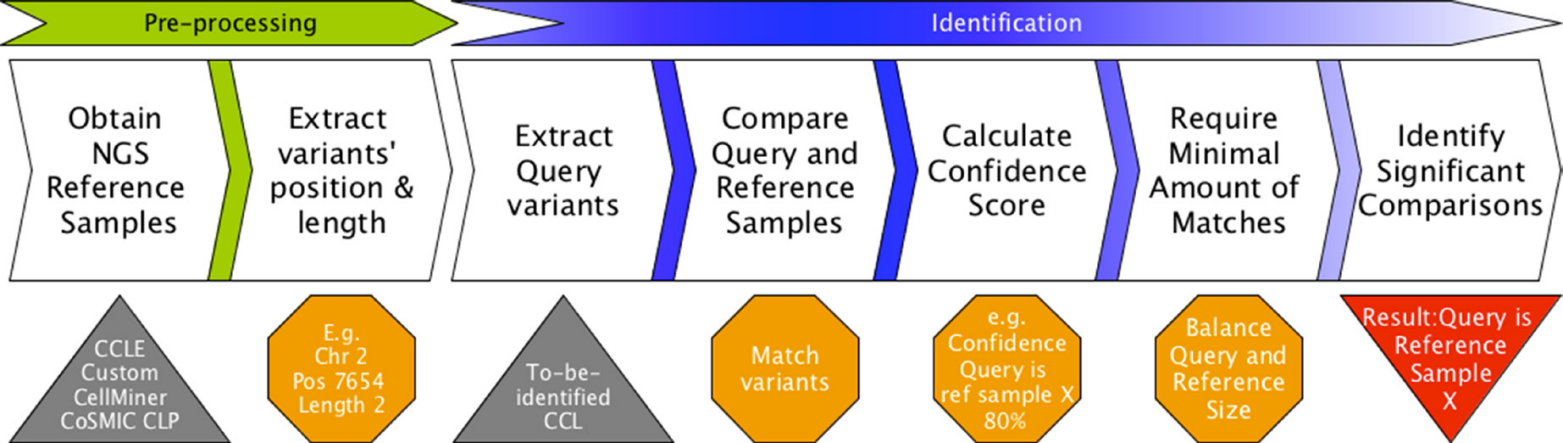
Uniquorn workflow CCLs from a reference library are compared to a given query sample q based on their set of small variants (variant profile). Variants are weighted according to their prevalence within the library (e.g. CCLE) and frequent variants are excluded afterwards. Subsequently, Uniquorn computes a confidence score quantifying the likelihood for each reference sample r being identical to q. Significantly different amounts of variants in q and r affect the statistical test that assesses whether q and r are similar. Therefore, a regularization step calculates the minimal amount of matching variants required to predict that q and r are related.

**Figure 2:**
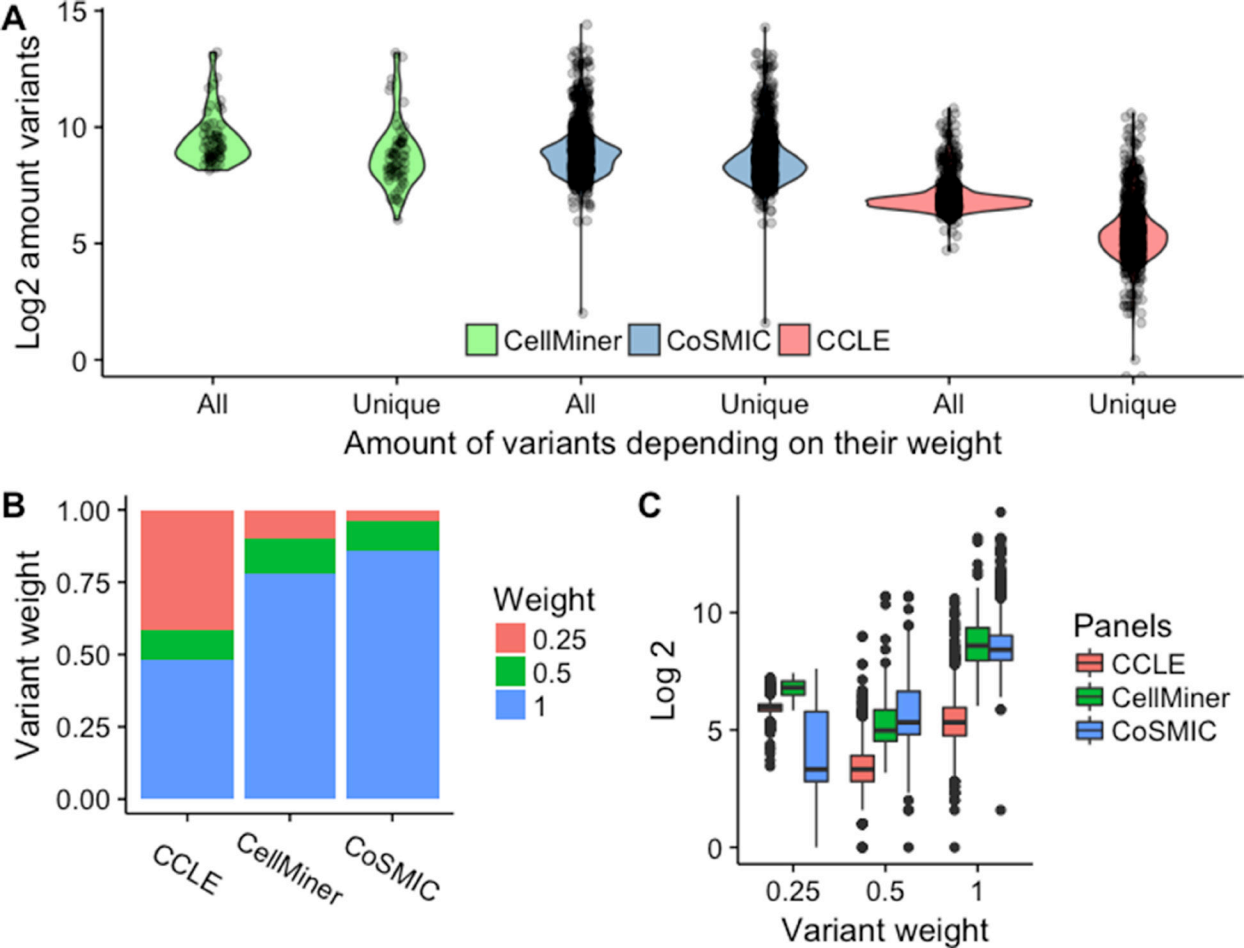
Distribution of CCL variant frequencies and weights across libraries (**A**) Number of “rare” variants in CCLs according to Uniquorn's weighting scheme. ‘All‘ shows the log-amount of variants per CCL without any filtering (weight 0.0) and ‘Unique shows the amount of variants that remain after all variants were filtered that were present in more than a single CCL (weight 1.0). Differences between software, technologies and filters (non-exhaustive) i.e. heterogeneous data-processing leads to different amounts of filtered, non-unique mutations as shown by the significantly different reduction of variants between the CellMiner (medium), COSMIC CLP (low) and CCLE panel (strong), see Table 3 for the sources of heterogeneity. It is shown, that all panels possess unique, i.e. ‘rare’ variants on which the Uniquorn identification method is based. (**B**) Distribution of weights per library. At least 50% of variants are high-weight (rare) variants. CCLE shows significantly less unique variants than COSMIC CLP and CellMiner, which explains the strong difference between raw and filtered variants in Figure A. (**C**) Number of variants per reference sample for different weight thresholds in the different reference libraries. CCLs from COSMIC CLP show a high amount of unique variants on average, especially when compared to those from CCLE.

The *Uniquorn* method identifies a query CCL by comparing its variant profile to that of all CCLs in a given set of reference libraries, see Figure 1. To this end, each variant in a reference library is weighted according to its inverse frequency. Only rare variants are used further. To assess the impact of different thresholds for this weight, we studied the distribution of variant counts in each of the three libraries (Figure 2A). As can be seen in Figure 2B, more than 50% of variants are unique within their library (weight 2 or higher), which means that even a very stringent threshold of 1.0 would filter out less than half of all variants. In Figure 2C, we show the distribution of the number of variants per CCL using different weight thresholds. When using only unique variants, CCLs from CCLE library have on average 153 variants in their profile (COSMIC: 744; CellMiner: 1139).

### Cross-Validation benchmark

**Figure 3:**
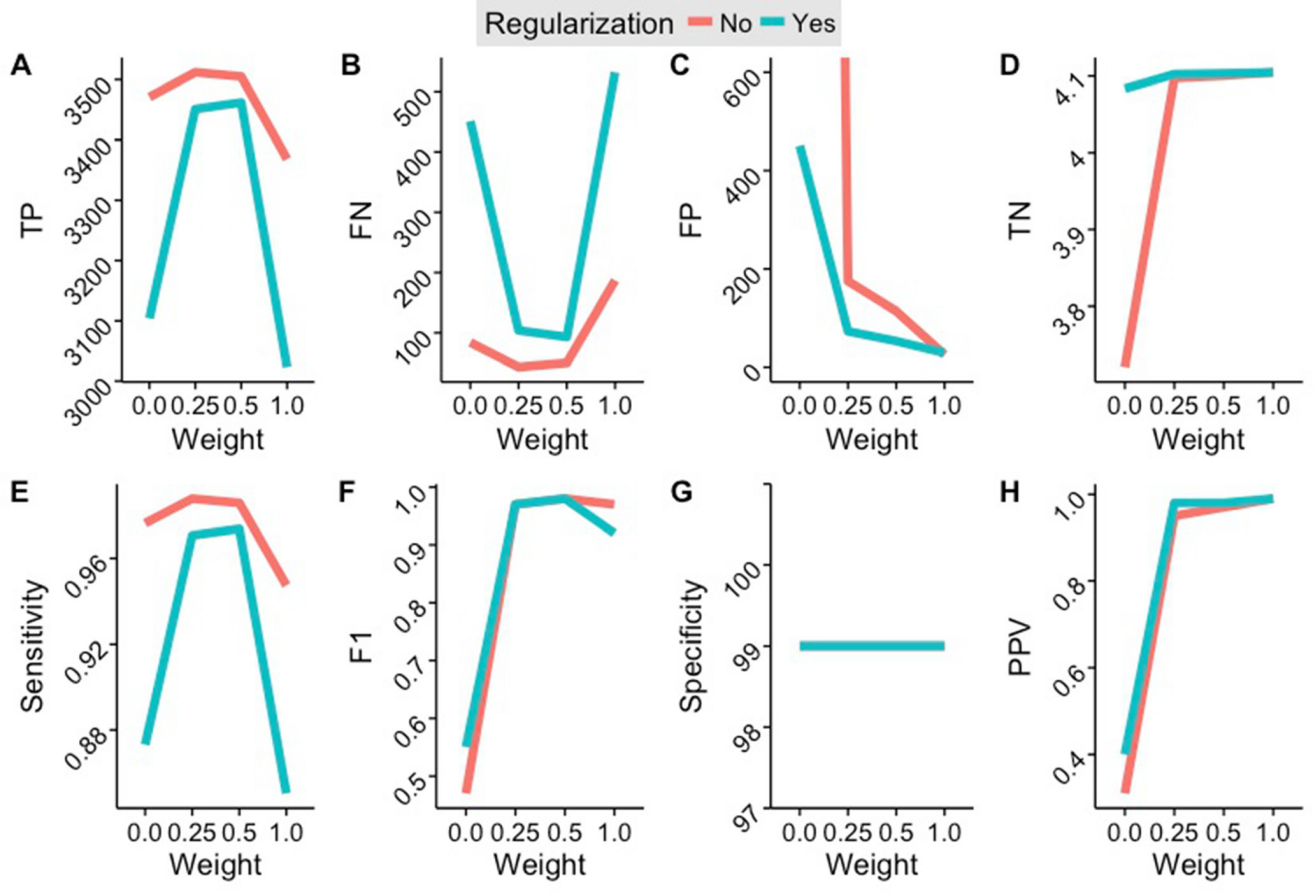
Results of the cross-identification benchmark depending on regularization and variant inclusion weight (**A**) Number of false positives. (**B**) Number of false negatives. (**C**) Number of false positives. (**D**) Number of true negatives. (**E**) Sensitivity. (**F**) F1-Score (harmonic mean of specificity and sensitivity). (**G**) Specificity. (**H**) Positive Predictive Value. Best specificity and sensitivity values are achieved using a weight threshold of 0.5. A threshold of 1.0 achieves the least false positives, most true negatives, and the highest positive predictive value.

We benchmarked the accuracy of Uniquorn using three large CCL libraries, namely COSMIC CLP, CCLE and CellMiner, which together embrace 1988 CCLs. We manually identified duplicates in this set and tested how reliably Uniquorn would detect them. To this end, each of the 1988 CCL samples was once utilized as query-sample and all three libraries as references. Since Uniquorn compares a single query-sample to all reference-samples, 1988 * 1988 ≈4E6 comparisons occurred during the cross-validation benchmark. Uniquorn predicted for each of the query-reference-pairs whether they were derived from the same cell line or not. Relative to the ~4E6 comparisons the amount of 3573 possible true positive identifications (1585 duplicates and 1988 self-identifications) is small which is why the positive-predictive value (PPV) is a particularly important evaluation measure. Results are shown in Table 1. The benchmark results show a very high specificity (at least 99%) across a range of weight thresholds, which can be explained by the extremely large number of true negatives. The more important metric is sensitivity, which is also very high for thresholds 0.5 and 0.25, correctly identifying 3474 and 3461 of the 3573 identical or related CCLs, respectively. Limiting the comparison to unique variants (weight threshold 1.0) yields the best PPV and lowest false positive rate (FPR), but lower weights of 0.5 and 0.25 result in higher sensitivity. Quantitative regularization slightly reduces identification efficiency, but supresses many false positive predictions. Figure 3 shows more detailed performance characteristics.

**Table 1:** Results of cross validation for different weight thresholds (columns 2 to 5)

Weight Threshold	1.0	0.5	0.25	0.0
**Maximally possible TPs**	3573			
True positives	3027	3474	3461	3111
	(3372)	(3521)	(3528)	(3485)
False negatives	546	99	112	462
	(201)	(52)	(45)	(88)
False positives	22	37	59	4631
	(18)	(94)	(155)	(7689)
Sensitivity %	85	97	97	87
	(94)	(99)	(99)	(98)
**Specificity %**	99			
F1 %	91	98	98	55
	(97)	(98)	(97)	(47)
Positive predictive value %	99	99	98	40
	(99)	(97)	(96)	(31)

A higher threshold enforces utilization of more specific variants but reduces the amount of considered variants. Depending on the threshold (0.0, 0.25, 0.5 1.0), between 3027 and 3474 of the 3573 true relationships between CCLs are successfully recovered. Numbers in brackets show results when the to-be-expected amount of matching variants is set manually to 10 variants; numbers without brackets show statistically estimated background-noise strength (regularized, see methods).

### Out-group benchmark

The previous evaluation measured the performance of Uniquorn when searching a CCL of a reference library within the set of reference libraries. We also tested how the method performs when it has to deal with profiles that are not derived from CCLs. Specifically, we used 1024 profiles from the 1000 genomes data set [21] as query samples and tested whether Uniquorn would assign them to a reference CCL – any such assignment certainly would be an error. Note that these comparisons work on very heterogeneous sequencing technologies, namely WGS-sequenced profiles (1000 genomes) with much smaller hybrid/exome-sequenced profiles (reference libraries). This implies large differences in terms of common polymorphisms (contained in 1000 genomes profiles, filtered in the references) and in the sheer number of variations (on average, a 1000 genomes profile consists of ~5E7 variations per sample compared to ~5E2 variations in the reference profiles). Using a weight threshold of 1.0 and regularization to cater for this difference, Uniquorn did not produce a single false positive prediction. These comparisons highlight the importance of our regularization step; omitting this filter, the comparison would produce 167 FP predictions for the ~2E6 comparisons.

**Figure 4:**
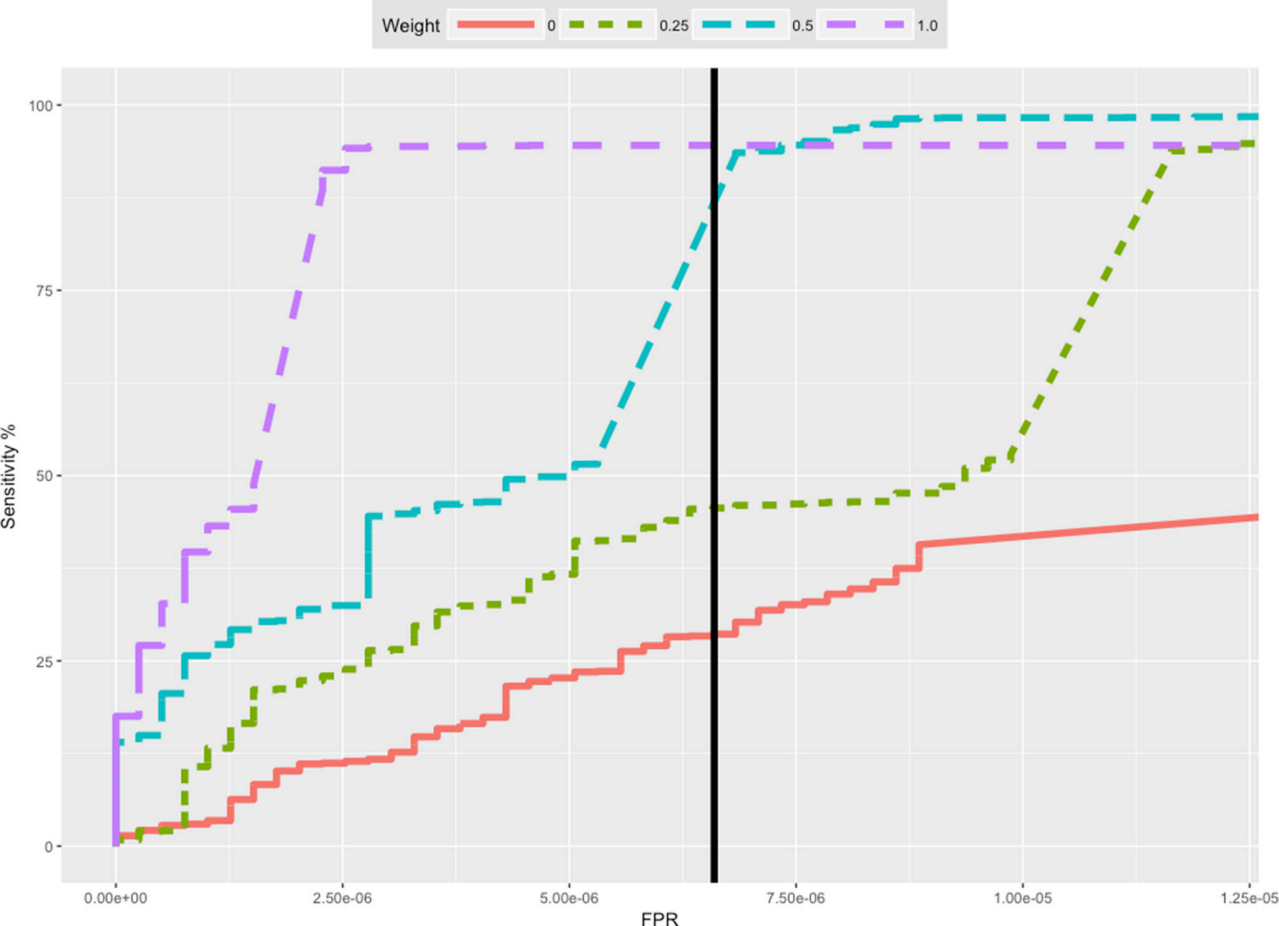
Receiver-Operator-Curves (ROC) of the cross-identification benchmark for different weight thresholds Thresholds 0.5 and 0.25 reached the maximal sensitivity (see also Table 1). The embedded plot shows the same ROC plot with an adjusted FPR-axis range to visualize the ROC curve of inclusion weight 0.0. The vertical black line shows the Uniquorn default threshold (confidence score of 10). The threshold was chosen as optimal cutoff between sensitivity and specificity.

Based on this and the previous experiments, Uniquorn's default confidence-score threshold is set to 10 [~ -log2(0.001)]. By default, the regularization filter automatically measures the strength of the background-noise and adjusts the required amount of matching mutations accordingly. However, users can set both thresholds manually to adapt to different reference libraries or to change the balance between false prediction rates and sensitivity (see Figure 4 for ROC analysis).

### Comparison to established methods

Uniquorn compares favourably to other methods for the identification of CCLs in terms of the amount of data and experimental work necessary (see Table 2). In first place, it is similar to established methods e.g. SPIA and Tandem-Repeat-Counting in that it is comparison-based. Uniquorn, however, is different to the aforementioned methods due to its focus on *in silico* identification of CCLs based on variant profiles obtained from different high-throughput sequencing technologies. Unlike SNP-based methods, Uniquorn does not depend on common, well characterized and publicly available genomic entities, but instead relies predominantly on rare somatic mutations, as SNP-based comparisons have severe drawbacks when applied in cancer research. First, SNPs with a minor allele frequency of > = 5% are frequently filtered from data sets (to focus on driver-mutations, e.g. by CCLE) and thus cannot be assumed to be generally available for CCL identification. Second, the loci of the most characteristic SNPs often are not genotyped during exome sequencing, and even less often so in panel sequencing. Moreover, cancer is frequently associated with large structural variants, often removing important loci, and with polyploid chromosomes whose variant calls cannot be directly compared to diploid references. Uniquorn was designed to robustly deal with such problems.

**Table 2:** Properties of Uniquorn compared to established methods for identification of CCLs

Identification Method for NGS CCLs	Physical sample required	Additional experiments required	Locus coverage required	Zygosity-pattern required	Dependent on reference genome
**STR (19)**	**X**	**X**	**-**	**-**	**-**
**SPIA (6)**	**X**	**X**	**X**	**X**	**-**
**NGS SNP (4)**	**-**	**-**	**X**	**X**	**X**
**NGS All Variants (Uniquorn)**	**-**	**-**	**-**	**-**	**X**

SPIA and STR require additional verification experiments on the physical CCL sample. Identification of CCLs by matching their SNP-zygosities directly from the NGS-data requires that the loci of the characteristic SNPs were sequenced and not filtered. For SPIA and NGS-SNP, zygosity calls have to be comparable (technology, ploidy, algorithms, etc.). Uniquorn only requires using the same reference genome for variation calling. Note that CCL samples created with a specific reference genome versions can be converted into another version, e.g. by a lift-over software, thereby decreasing the severity of this limitation.

We also compared identification results of Uniquorn and the SNP-based method by Demichelis et al. [4] quantitatively. 130 of the 155 CCLs used by Demichelis and colleagues are present in the Uniquorn benchmark set. These 130 CCLs have 265 different representations in our data set because many are present in different CCL reference libraries. Uniquorn identified 100% of these 265 CCLs at an inclusion weight of 0.5 (see Supplementary Material File 3). Thus, Uniquorn showed an equal performance compared to the established SNP-based identification methods.

## DISCUSSION

### Analysis of Mis-classifications

Analysis of the 22 false positive (FP) predictions from Table 1 (weight 1.0) revealed that all FP-predictions were caused by a set of only 13 CCLs. These CCLs have in common that their profiles are very small; they have a mean size of 366 (sd = 4E3) variants, while the profile sizes of CCLs that were never predicted as FP have a mean size of 3768 (sd = 8E2) variants (*p* = 0.006). 20 of these 22 FPs occurred with a query sample identifying a reference from a library which does not contain the query, which means that they would not occur if a lab can safely exclude a reference library from considerations. The most problematic CCL regarding FP is *HCC-2998*, which is contained in CellMiner and COSMIC CLP. Accordingly, it was used twice as query, and produced five FP in total (3 FPs when used as query and 2 FPs when used as reference). When used as query, *HCC-2998* correctly identified itself in CellMiner and COSMIC CLP with high confidence. However, it was also predicted to be similar to three CCLs from CCLE (*JHUEM-7*, *SNU-81*, *HEC-251*). These false predictions all had very low confidence scores, sharply above the threshold, and can be explained by to the stronger influence of randomly matching variants within small profiles.

Three factors have been found to be associated with false negative (FN) predictions: About 100 of the 546 FN-predictions for weight 1.0 occurred between query-reference pairs that were defined as identical by the gold-standard due to either cross-contamination (e.g. *ACCS* and *T24* [7]) or an origin within the same human being but not the same cancer-tissue (e.g. *AU-565* and *SKBR-3* [22]). Secondly, FN predictions are enriched in CCLs with small profiles. CCLs that failed at least once to identify a related query have on average 345 (sd = 2E2) variants, while CCLs that always identified their counterparts successfully have on average 528 (sd = 1E3) variants (*p* = 1E-8). Thirdly, CCLs that are highly similar to another CCL within the same library generally perform poorly because in those cases the amount of rare variants is insufficient. For instance, *HEL* and its closely related sub-clone *HEL92.1.7* [23] both failed to identify themselves because they are so similar that none of their variants is unique within the library. This effect can be diminished by appropriate adjustment of the weighting scheme, as can be seen by a FN-reduction of 82% from weight 1.0 to weight 0.5. However, these cases are rare within our evaluation data: As shown in Figure 2, unique variants are present in 1986 out of 1988 CCLs (99.9%).

### CCL-identification based on generic ‘omics-sequencing data

Every NGS technology that allows calling of small genomic variants could, in principle, be utilized to identify CCLs based on the Uniquorn method. We believe that bulk-RNA-seq should be utilizable without conceptual changes, although we did not yet test our algorithm with such data. Panel-seq will at least require the re-adjustment and optimization of thresholds to compensate for the relatively low number of variants. Furthermore, since fewer matching entities may already indicate that two CCLs are similar, the statistical tests for matches occurring just be chance might have to be strengthened. Usage of single cell technologies would require adjustments to compensate for higher impact of random events (noise). Less similar NGS technologies, such as methylation, Chip-seq or Atac-seq, probably would require more profound changes to our method.

## MATERIALS AND METHODS

### Reference libraries

Uniquorn compares NGS data of a given query sample q with that of samples r from a given CLL library R. Currently, three large libraries are integrated into the package: (1) COSMIC CLP, obtained January 13^th^ 2016 from http://cancer.sanger.ac.uk/cell_lines (2) CCLE, obtained January 13^th^ 2016 from http://www.broadinstitute.org/ccle and (3) CellMiner, obtained January 13^th^ 2016 from http://discover.nci.nih.gov/cellminer. All data sets are based on the same reference genome HG19/ GrCH37. Variant profiles and CL-names were directly parsed from the files provided. Note that the Uniquorn package also features an API for adding novel, possibly in-house-created, reference libraries.

Table 3 shows most important characteristics of the three libraries. COSMIC CLP is the largest data set with 1024 whole-exome genotyped CLs from 30 tissues. CCLE contains 904 hybrid-capture genotyped CLs from more than 36 tissues. The CellMiner project comprises whole-exome genotype data of the NCI-60 panel from 9 tissues.

**Table 3:** Characteristics of the three CL reference library used in this work

Reference Library	Total number of variants	Cancer Cell Lines	Ø Variants per CL	Number of genes covered	Variant calling software	SNP MAF filtering
COSMIC**CLP**	760E5	1024	7,4E5	20965	Caveman(13)	> 0.0
					Pindel (14)	(all)*
**CCLE**	140E5	904	1,5E5	1651	MuTect (15)	> = 0.05
**CellMiner**	0,68E5	60	0,01E5	> 20 k	GATK (16)	None

The absolute and the average number of variants differ by orders of magnitude since different technologies and algorithms were utilized for sequencing and variant calling. Moreover, the number of genes covered varies strongly. SNPs – required for SNP-based identification - have been mostly or completely excluded in two of the three sets. For the COSMIC CLP, two different methods were used to call small variants and indels.

### Confidence scoring

Uniquorn represents each sample (query or reference) by its variant profile, which is defined as the sequence of substitutions or small insertions and deletions compared to the reference genome. Each variant is encoded by its start position and its length. The scoring of query and reference samples is library-specific, i.e., the score obtained from the comparison of query q with a sample r from reference library R assesses the likelihood that q is identical to r independently of all other libraries. This reflects the fact that in a typical setting the set of potential contaminators, i.e. all samples from which q could have been derived in principle, is known.

When comparing query q to a reference sample r, Uniquorn estimates the likelihood that their profiles stem from the same cell line. Developing a complete model for assessing this likelihood would require exact knowledge about the ways how the profiles of q and r were obtained, i.e., the error rates and distributions of the sequencing technologies applied and of the entire variant calling procedures. Since such detailed data is not available for most techniques, we developed a simple yet highly effective heuristic for quantifying the likelihood of identity using only variant profiles (see Figure 1). The algorithm first weights all variants found in R according to their frequency. In a second step, it discards variants whose weight is below a given threshold. Next, Uniquorn computes the overlap in remaining variants in q and each r and derives a multiple testing corrected *p-value* for the likelihood that these sets stem from the same cell line. This likelihood is based on the assumption that the profiles of q and r have the same sequencing scope (panel, whole exome, whole genome), although the sequencing technology used might have been different. For the case that different sequencing scopes were applied for q and r, which will result in a strong difference between the numbers of variants found, we compute a second threshold taking into account the spread of randomly matching variants between q and all r. Each of these steps is explained in detail in the following paragraphs.

### (1) Variant weighting

As preparatory step, each variant v found in any sample of the given reference library R is weighted according to its frequency f_v_ using:

$$w\left(v\right){=2}^{-(f_{v}-1)}$$

Variant weights are library-dependent, i.e., the same variants will receive different weights in different libraries to reflect the inherent divergence of sequencing technologies and algorithms. Uniquorn identifies samples by their characteristic variants, i.e., variants with a high weight. The default threshold is 0.5, i.e. the further scoring considers only variants occurring maximally two times in one respective reference library R. Other thresholds can be chosen as well, depending on the desired trade-off between sensitivity and false positive rate (see Table 1 and Discussion).

### (2) Confidence score calculation

After filtering non-characteristic variants, q is compared to all reference samples from R to obtain a pair-wise confidence score. To this end, we model CCL profiles as a set of variants drawn at random from the set of all variants in R and assess the probability of the overlap of variants in q and in each r using an overrepresentation test. Let T be the number of variants in R, N be the number of variants in r, n the subset of these also found in q, and k = N-n the number of variants in r not found in q. Then, the probability of a given variant in r to also occur in q is p_r_ = N/T. Accordingly, the likelihood to miss exactly k variants from r in q is

$$D_{r}^{k}=\left(\begin{array}{c} N\\ k \end{array}\right)q_{r}{}^{k}p_{r}{}^{N-k}=\frac{N!}{k!\left(N-k\right)!}{\left(1-p_{r}\right)}^{k}p_{r}{}^{N-k}$$

Following [24], we next compute a *p-value* ${\hat{p}}_{r}$ by summing up the probabilities to miss up to k variants.

$${\hat{p}}_{r}=\sum_{N-n=k}^{N}D_{r}^{k}$$

${\hat{p}}_{r}$ is the probability to commit a statistical error of type one when rejecting the null-hypothesis *H_0_*, which here states that the k variants missing in q with respect to r are missing because r and q are different CCLs. The *p-values* are corrected for multiple testing with the Benjamini-Hochberger method [25]. We use the negative logarithm of the corrected value as the confidence *c_r_* that q and r represent the same cell line. We put a threshold on this score which is determined empirically by balancing sensitivity and specificity in our test data (see the ROC-curve in Figure 4).

### (3) Quantitative regularization

The confidence score derived above is based on the model that q's and r's variant profiles were both created by randomly drawing n variants from R. This implies that both profiles are of roughly the same size, a valid assumption when both profiles were obtained using the same sequencing scope. However, if the number of variants in q is much different from the average number of variants in samples from R, this assumption is most likely wrong. This occurs, for instance, if samples in R were panel-sequenced while q was whole-genome sequenced, or if both were whole-genome sequenced, yet all known SNPs were filtered from the profiles in R. In such cases, our confidence will be either overly optimistic (large variant overlap due to a much larger profile of q) or overly pessimistic (low variant profile due to a much smaller profile of q). For correcting for such cases, we model the estimated number of additional variants between q and any r through a spuriousness variable *sp, sp* ε [0,..,1]. is estimated by the integral of the beta function with parameters s_max_ and s_mean_, where s_max_ is the maximal number of shared variants between q and any sample from r, and s_mean_ is the mean of the number of these matches. The beta function has been found to suitably estimate the expected number of additional variants in that it is governed (1) by the relative number of matches and (2) by the absolute size of its input-parameters and (3) by its boundedness to [0,..,1]. Thereafter, a threshold *M_min_* on the acceptable amount of observed unmatched variants is calculated as

$$M_{min}=\frac{S_{mean}+S_{max}*SP}{\left(1-SP\right)}$$

If the confidence score threshold and the *M_min_* threshold are met, the variant profile of a reference CCL r is predicted to stem from the same cell line as the profile of q. Note that this implies that multiple cell lines from the same reference library might be predicted to be identical to q. We find this strategy to have advantages over the option to simply return the best matching reference sample, as we thus (1) do not have to assume a reference library to be duplicate-free, and (b) may also report that none of the reference cell lines to be identical.

### Evaluation

We benchmarked Uniquorn using all 1988 CCLs from the three data sets described above (see Table 1) as query sample against each of the three reference libraries; thus, we performed 1988*1988 ~ 4E6 comparisons in total. A true positive identification was counted when Uniquorn predicted that a query was identical to a reference CCLs in accordance with a gold standard (see below); analogously for true negatives. A false positive was counted when Uniquorn predicted query and reference CCL to be identical but not the gold standard. False negatives were cases were query and reference CCLs were assessed as not being identical by our algorithm but identified as such in the gold standard.

**Figure 5:**
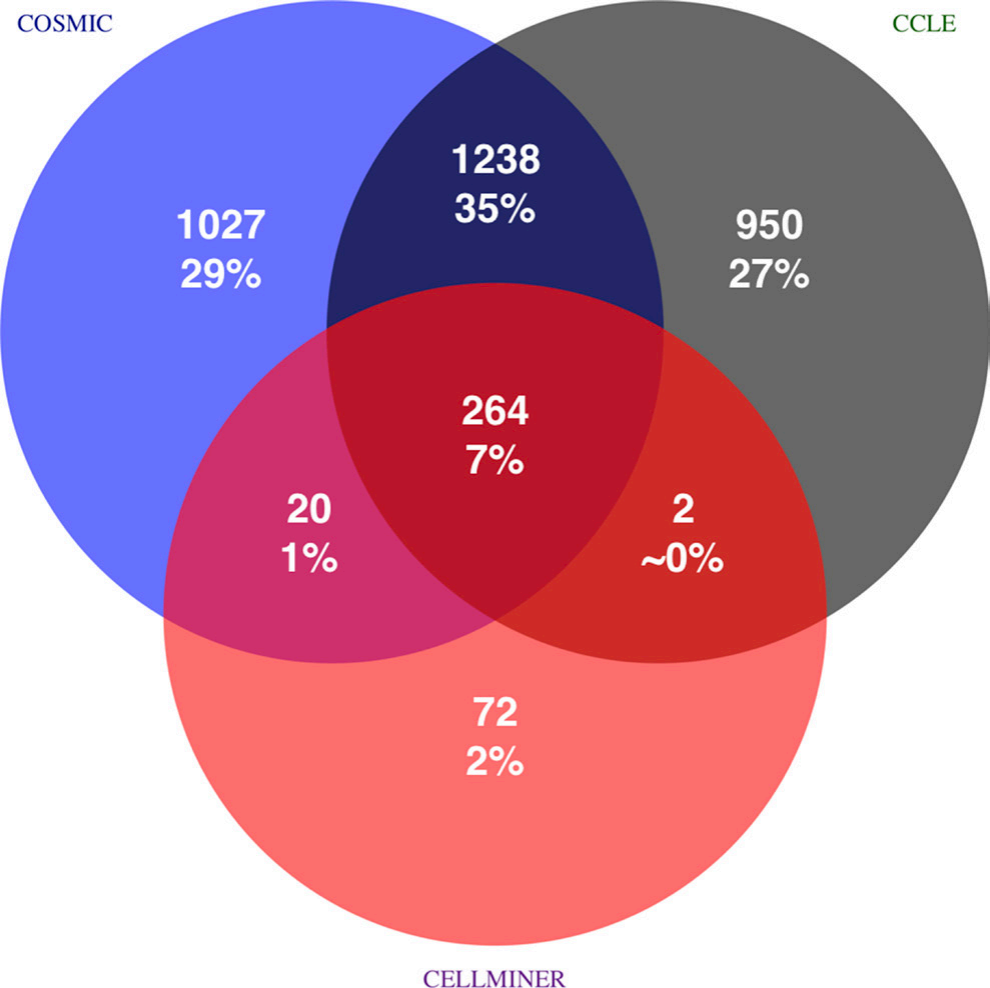
Source of true positive CCL identifications based on gold standard Total amount, percentage, and source of all 3573 TPs for each of the 1988 CCL samples are shown. For instance, 1238 TPs are identified because copies of the same or highly similar CCLs are contained in COSMIC CLP and CCLE. Positive identification within a single circle are due to relatedness of CCLs within the same library and self-identifications. 43% of all possible true positive cross-identifications are due to CCL copies in different reference libraries. Percentages do not sum up to 100% due to rounding errors.

Note that the maximal number of true positives (TP) per query in this evaluation scheme depends on whether this CCL was present in only one or in more than one data sets (many such cases exist; see Figure 5). If a CCL exists only in a single reference library, only one TP can occur. If it is part of two libraries or has related identified CCLs within the same library, four TPs are possible, since each will be used as query and should identify both itself and the related sample; for CCL in all three libraries, maximally nine TPs can be found. Using our gold standard, a maximum of 3573 TPs is possible.

### Gold standard set creation

The gold standard defines which pairs of CLs are considered identical within our evaluation. To create a gold standard we first defined all CCLs with the same regularized name as identical. CCL names were regularized by removing any non-alpha-decimal and capitalization of all remaining characters. In a second step, we manually confirmed or rejected the identity of all CCLs whose names only differed by a small prefix or suffix, such as *MDA-MB-435* and *MDA-MB-435s*. In a third step, we screened the literature for cases were CLs with same regularized name were reported as being different, e.g. *TT* and *T.T*, and adapted the gold standard accordingly for these cases. A list showing the CCLs that were defined as similar as a result of the literature screening can be found in Supplementary Material File 2. Note that pairs of identical CCL may be part of the same or of different reference libraries (See Figure 5).

After the evaluation, we furthermore checked all FP predictions to see if these are indeed FPs or errors in the gold standard (see Discussion); one such example is the pair *SNB19* and *U-251*, which have completely different names but denote the biologically identical CCL [4]. The entire gold standard is available in Supplementary Material File 1.

### Implementation and availability of data sets

The method was implemented in the freely available R-Bioconductor package *Uniquorn*. The benchmark libraries CCLE and COSMIC CLP can be freely obtained and used as Uniquorn reference libraries. The CellMiner Project library is included by default. Custom libraries can be created e.g. for identification of proprietary CCL samples.

## CONCLUSIONS

Uniquorn is a novel *in-silico* method for helping to avoid confusion of cancer cell lines during lab processing. Specifically, it compares the mutation profile of a given query CCL to those of CCLs in reference libraries to identify all cell lines from these libraries that are genetically suspiciously similar to the query. Compared to existing methods for CCL identification, Uniquorn works across a range of sequencing techniques and can also be applied after SNP filtering; furthermore, assuming the CCL today are anyway sequenced in most projects, it does not require any additional experimentation. The software is freely available and can easily be adapted to specific reference libraries or specific requirements regarding specificity and sensitivity of the results. Uniquorn has been benchmarked by cross-identifying 1988 CCL samples from three different providers, using different sequencing technologies. A sensitivity of up to 97% and specificity of 99% has been achieved. In future work, we plan to adapt Uniquorn to also robustly identify profiles obtained from gene panel sequencing and RNA-sequencing.

## SUPPLEMENTARY MATERIALS FIGURES AND TABLES








