# Supplementary material for: Robust in-silico identification of cancer cell lines based on next generation sequencing
Source: Oncotarget. 2017 Mar 10;8(21):34310–20. doi: 10.18632/oncotarget.16110 (PMC5470969; doi:10.18632/oncotarget.16110)
Supplement: Supplementary file 1 [file oncotarget-08-34310-s001.pdf]

Robust *in-silico* identification of cancer cell lines based on next generation sequencing

Supplementary Materials

Supplementary Material File 1: Gold-standard that defines which CCLs relationship predictions are correct for the 1988 benchmarked CCLs. See Supplementary_Material File_1

Supplementary Material File 2: List of known relationships between the 1988 CCLs and the reason for the relationship. Used for creation of the Gold-standard. See Supplementary_Material File_2

Supplementary Material File 3: Excel file that lists all benchmark-identification results for all 1988 samples, both regularized and non-regularized for every inclusion weight. See Supplementary_Material File_3
